# A conversation on technology transfer

**DOI:** 10.1038/s41467-022-30885-5

**Published:** 2022-06-08

## Abstract

This Q&A about technology transfer is intended as a useful resource to the *Nature Communications* readership, particularly academic scientists working in the life and physical sciences who have an interest in commercializing their research. We spoke to Dr. Andrea Crottini, Head of the Technology Transfer Office at the École Polytechnique Fédérale de Lausanne, who provided insights into the possible avenues to pursue.

**Figure:**
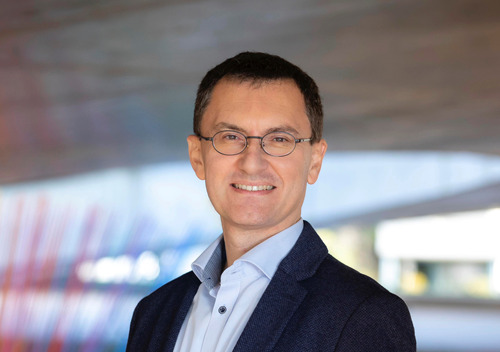
Dr. Andrea Crottini

As a technology transfer office, you serve as a bridge between academia and industry, assisting scientists in commercializing their technologies. Based on your experience, can you tell us what makes a good candidate for commercialization? What criteria do you use to assess suitability?

Contrary to what might be expected, the main factor is not necessarily the idea or technology itself, but people’s involvement. The actual and future commitment of the individuals involved in the commercialization of the technology is paramount, both on the academic and industrial sides.

The commercialization of technologies is a long journey, from development, through de-risking, including prototyping and preliminary clinical validations, market analysis and industrialization, to the first sale. As no technology will find the path to commercialization by itself, long-term commitment is key.

On the academic side, the researchers and technology transfer office (TTO) must be convinced about the technology and, in turn, be able to convince potential licensees. It is therefore important to regularly discuss the technology within the university, to challenge both researcher and TTO beliefs and commitments with the main questions being: What is the actual purpose of the technology? And is it still the best technology serving this purpose?

The early assessment of the market potential is another important factor, although many technologies may not fulfill a clear market need upon first analysis. In the field, we say that at least a certain degree of pain point, meaning an unsolved and pressing problem, must exist in potential customers to positively consider a technology in view of commercialization.

A third criteria is the maturity of the technology itself. As any new business is risky, of course a ready-to-be-used technology would be preferred. That being said, early-stage, high risk solutions should not be excluded. This is why the commitment of researchers is again important: they will bring the technology to maturity alongside the tech transfer process!

What should researchers who wish to start their own companies do to develop their technology, if they are not interested in finding industrial sponsors? Is there a step-by-step guide?

Entrepreneurship requires, above all, personal interest and motivation, as the researchers will become the main actors guiding the success of the technology.

As I mentioned earlier, technology transfer requires long term commitments in academia and industry. Entrepreneurship is an effective way to increase the odds, by having a single actor transitioning and playing both roles. While this strategy requires a double commitment in terms of time and risk taking, it may lead to a higher potential reward for the researcher.

There is no step-by-step guide that works for every start-up. The best definition of a start-up is probably Steve Blank’s “a temporary organization designed to search for a repeatable and scalable business model”^1^. As such, each start-up must determine its own way of operating, and there is no specific handbook for this. Of course, a significant amount of time is dedicated to procuring the funds that will be necessary to bring the idea to market. In fact, one metric to measure start-up success is to look at how much money it has raised.

It’s certainly a positive development that PhD students and postdocs now have a third option to consider besides staying in academia or taking a job in industry — that of becoming an entrepreneur — and an increasing number of great examples of entrepreneurs and start-up role models exist. The inspiration and advice they provide are probably more effective than any book reading. Great entrepreneurs create a virtuous environment: in locations where they have successfully operated, entrepreneurship in new generations will self-ignite. That is not to imply that one has to necessarily share the same office as a thriving entrepreneur to be inspired — many stories of success have entered the mainstream through the news and even movies, which can encourage aspiring entrepreneurs.

In the usual scenario, researcher-entrepreneurs are early-birds, as they will catch their technology for commercialization before companies even know about it. However, we have also seen start-ups and existing companies competing to license the same technology, as well as successful technology transfer to start-ups after failure to license to existing companies. There are plenty of possibilities!

When would you recommend creating a spin-off or start-up company? What are the associated advantages and disadvantages? And what are the alternatives?

If personal motivation and commitment to entrepreneurship are present, the start-up route is the way to go. It’s important to understand that many TTOs do not create start-ups. Researchers, as “founders”, do it.

Determining the “when”, meaning the right moment to create a start-up, is a challenge. The time window dictated by the time-to-market of the technology or the time required to bring it to appropriate maturity, and the time frame in which the public will actually need that technology should, in principle, provide the timeline of the business. But in practice this is a crystal ball exercise and faster time to market is not always better — the timing must be right, as some historic examples have taught us. For start-ups, the capacity to adapt to market changes and any relevant regulations will be most important.

One caveat exists for start-ups developing technologies that will clearly address only niche markets. In this scenario, creating a company structure for a small business might involve substantial effort without paying off. Investors and publicly available funders may also be unsupportive. In this case, an existing company, instead of a start-up, might be a better candidate to develop a product serving a rather small market, as the business structure is already in place.

Licensing to existing companies is always a valid alternative to start-up creation, for example in the absence of entrepreneurs.

So what happens when the researcher is not interested in becoming an entrepreneur, but would nevertheless like their idea to become commercialized? Is there a model in which the researcher can approach an industrial partner and share the responsibilities and development costs, and eventually the revenues, if profitable?

In this case, the TTO licenses the intellectual property (IP) to an existing company. In general, exclusive licenses work well. The company will enjoy exclusivity on the IP, will take over all future IP costs, and will pay royalties to the university depending on the commercial success. Royalties are percentages of the sales of the licensee products covered by the IP. In many universities, a share of these royalties will be distributed to the inventors, hence resulting in financial reward for them!

We’d now like to walk through the three steps typically associated to bringing a technology to market in the academic sphere: filing an Invention Disclosure, securing Intellectual Property Rights, and licensing, starting with the first one. Could you tell us what Invention Disclosure means and when it is required? And what happens after disclosure?

An Invention Disclosure (ID) is a written declaration to the university made by inventors consisting of a detailed description of a new and inventive technology, with respect to known approaches and solutions, and its potential uses. It also includes the complete list of inventors and possible contracts related to the new technology, like collaboration agreements with third parties or external funding.

The ID then allows one to start the process of patent evaluation, which is typically considered when the technology has the potential to be taken on by a company. A patent is a proprietary title related to an invention, and the ID provides all the details about the content of the invention, the inventors, and the initial owner(s) of the invention.

What are Intellectual Property Rights and when should one consider applying to protect them? What is needed in order to do so?

The various legislations recognize the ownership rights of objects, but also of some immaterial, or intangible items, produced by the human intellect. This is the case for original creations, like inventions, literary works, unique names of goods or companies, designs and others.

Nations recognize and award the immaterial ownership with specific titles, or IP rights: patents for inventions, trade-marks for commercial names, design rights for aesthetic creations, copyrights for literary and software works. The majority of technologies are licensed as patented inventions or copyrighted software.

The three common questions to be asked when considering the protection of IP rights in technology are: “Is it new?”, “Is it original or inventive?” and “Is it useful?” If the answers are yes, then the registration office in the respective country will be happy to receive and examine the IP rights!

And finally: what is a license and what happens post-licensing?

A license is the permission granted by the owner of the IP rights — be it for example a patent, software or a trademark — to a third party, the licensee. A license is a written agreement, which includes a negotiated economic consideration for the owner, in the form of fees or royalties, paid by the licensee for the use of these rights. The license can be exclusive or not, and will also encompass some performance obligations for the licensee, in terms of technology development and commercialization milestones.

Post-licensing activities include regular exchange of information between licensor and licensee about the technology status and sales. In case of start-ups, when the university obtains shares in the company as financial return, post-licensing will also comprise the university shareholding activities.

Thank you so much for taking the time to talk with us and sharing your insights into technology transfer!
